# Detection of Free-Living Amoebae and Their Intracellular Bacteria in Borehole Water before and after a Ceramic Pot Filter Point-of-Use Intervention in Rural Communities in South Africa

**DOI:** 10.3390/ijerph18083912

**Published:** 2021-04-08

**Authors:** Clarissa van der Loo, Catheleen Bartie, Tobias George Barnard, Natasha Potgieter

**Affiliations:** 1 Water and Health Research Centre, Faculty of Health Sciences, University of Johannesburg, Johannesburg 2094, South Africa; clarissakruger@gmail.com (C.v.d.L.); tgbarnard@uj.ac.za (T.G.B.); 2 CB Scientific, Roodepoort 1724, South Africa; delene.bartie@cbscientific.co.za; 3 Environmental Health, Domestic Hygiene and Microbial Pathogens Research Group, Department of Microbiology, University of Venda, Thohoyandou 1950, South Africa

**Keywords:** amoeba-resistant bacteria (ARB), borehole water, ceramic filter, free-living amoeba (FLA), point-of-use intervention, rural communities

## Abstract

Free-living amoebae (FLA) are ubiquitous in nature, whereas amoeba-resistant bacteria (ARB) have evolved virulent mechanisms that allow them to resist FLA digestion mechanisms and survive inside the amoeba during hostile environmental conditions. This study assessed the prevalence of FLA and ARB species in borehole water before and after a ceramic point-of-use intervention in rural households. A total of 529 water samples were collected over a five-month period from 82 households. All water samples were subjected to amoebal enrichment, bacterial isolation on selective media, and molecular identification using 16S PCR/sequencing to determine ARB species and 18S rRNA PCR/sequencing to determine FLA species present in the water samples before and after the ceramic pot intervention. Several FLA species including *Acanthamoeba* spp. and *Mycobacterium* spp. were isolated. The ceramic pot filter removed many of these microorganisms from the borehole water. However, design flaws could have been responsible for some FLA and ARB detected in the filtered water. FLA and their associated ARB are ubiquitous in borehole water, and some of these species might be potentially harmful and a health risk to vulnerable individuals. There is a need to do more investigations into the health risk of these organisms after point-of-use treatment.

## 1. Introduction

Free-living amoebae (FLA) are ubiquitous in the natural environment (in biofilm; in water–soil, water–air, and water–plant interfaces) where they play an important part in nutrient and energy turnover by acting as bacterial population-controlling predators [1]. Although FLA are known for their contribution to plant growth, nutrient cycles, and soil mineralisation by feeding on microbes [2,3,4], several studies have also reported on the isolation of FLA from anthropogenic ecosystems, such as cooling towers, air-conditioning units, tap water, hospital water reservoirs, and humidifiers [5,6,7,8,9].

Free-living amoebae are part of a large polyphyletic group, with organisms ranging from non-pathogenic to pathogenic in humans [2,10]. Pathogenic FLA often lead to brain pathologies [11,12], such as granulomatous amoebic encephalitis (GAE) caused by *Acanthamoeba* spp. and *Balamuthia mandrillaris,* and primary amoebic meningoencephalitis (PAM) caused by *Naegleria fowleri*—many of these infections are fatal [13,14]. Healthy individuals are also at risk, with FLA colonising throats and nasal tracts [15,16]. *Acanthamoeba* spp. and *B. mandrillaris* have been isolated from both skin and lung infections [11], *Entamoeba histolytica* (an enteric parasite) can cause amebiases, or liver abscesses [17] and is responsible for up to 100,000 deaths annually [18,19], and *Sappinia diploidea* has been found as causative agent in encephalitis in a healthy young male [20].

*Acanthamoeba* spp. are classified by genotype, and based on 18S rRNA sequencing, there are currently 17 genotypes identified, each with 5% or more divergence between the different genotypes [21]. The genotypes include T1-T12 [22], T13 [23], T14 [24], T15 [25], T16 [26], and T17 [27]. Many human infections can be associated with the T4 genotype [28]. Moreover, non-pathogenic FLA species are dangerous for human and public health as they are known hosts for pathogenic bacteria [7,29,30]. For example, *Vermamoeba vermiformis* (previously known as *Hartmanella vermiformis*) is a well-known FLA host for amoeba-resistant bacteria (ARB) [31], especially *Legionella pneumophila* [32].

Amoeba-resistant bacteria such as *Legionella* spp., *Mycobacterium* spp., *Escherichia coli*, *Salmonella* spp., *Shigella* spp., *Pseudomonas aeruginosa* and *Listeria monocytogenes* can resist amoebal microbiocidal mechanisms and are able to survive amoebae encystation, allowing them to grow intracellularly while being protected from unfavorable conditions until the conditions improve and the amoebae excyst [29,33]. A review by Thomas et al. [7] listed a total of 102 bacterial species such as *Achromobacter* spp., *Klebsiella* spp., *Mycobacterium* spp., *Legionella* spp., *Stenotrophomonas* spp., *Enterobacter* spp. and *Pseudomonas* spp., to name a few, which were described as “surviving and/or flourishing” when in contact with various FLA species. These ARB species are all human pathogens [34,35]. Studies have shown that passage through an amoebal host causes the adaptation of some bacterial species to survive inside human macrophages [36,37]. Other studies have shown that ARB species isolated from nasal mucosa in humans have adapted to intracellular life and spread to the lower respiratory tract where it caused disease [36].

There are different groups of ARB: (1) facultative intracellular (the most abundant), (2) obligate intracellular, and (3) extracellular [29]; examples of each include *L. pneumophila* in many FLA species [38], *Mycobacterium leprae* in *Acanthamoeba* spp. [39], and *Vibrio cholerae* in *Acanthamoeba* spp. and *Naegleria* spp. [40], respectively. A symbiotic relationship may evolve if the amoebal cell’s defense mechanisms are either impaired or inefficient, allowing the bacteria to persist internally [2]. Infections caused by ARB are diverse, ranging from life-threatening pneumonia in patients on artificial ventilation, cystic fibrosis [41], and chronic granulomatous disease [42]; to outbreaks of skin infections following liposuction [43]; furunculosis after domestic footbaths [44]; mastitis after body piercing [45]; and abscess formation in people getting intravenous injections with alternative medicine and used vials contaminated with *Mycobacterium abscessus* [46].

Rural communities in South Africa generally do not have access to treated drinking water and must rely on any available water sources, which include boreholes [47,48]. The households use these water sources for their drinking, cooking, and other household needs. In many instances, this water is consumed without any form of treatment that have been shown to prevent and reduce diarrhoeal- and other waterborne diseases [47,48,49]. Potgieter et al. [50] and Taonameso et al. [51] have shown that borehole water used by rural communities is a potential health risk to rural communities if not treated adequately before drinking by the household members.

Potters for Peace (PFP) designed a low-cost ceramic filter and assisted in the production thereof throughout the world (www.pottersforpeace.org, accessed on August 2020) as a water treatment system to reduce diarrhoeal diseases. The ceramic filter (shaped like a coned flowerpot) can hold 7.1 litres of water and fits inside a 20-litre plastic receptacle with lid and spigot [52]. Several studies have showed that not all point-of-use filters following the Potters for Peace design and method are created equal [52]. During the production, clay is mixed with different types of husk (depending on the availability in each country) and baked; the high temperature of the ovens causes the husks to burn away and leave small pores through which the water can travel and, essentially, trap microorganisms. Due to the nature of the filter construction, the pore sizes are not always uniform, as the sawdust in the clay burns away in the firing process—for this reason, there is colloidal silver painted on the inside of the filter to add to the disinfection capabilities of the system [52]. Some devices have been tested in controlled environments, such as laboratories, and others are evaluated in the field [49]. Generally, the effectivity of these filters in the field during intervention studies to stop the spread of diarrhoeal diseases is tested using only indicator organisms such as total coliform bacteria and *Escherichia coli* (*E. coli*) counts per 100 mL [52].

Therefore, the purpose of the study was to isolate and identify FLA and ARB from borehole water used for human consumption in rural households before and after a PFP ceramic pot filtration device as an intervention.

## 2. Materials and Methods

### 2.1. Study Consent

An adult representative from each participating household gave consent by signing a consent form that was administered by a trained member of the research team who translated and explained the study process to the participant. Study participation was completely voluntary, and participants could withdraw from the study at any time.

### 2.2. Study Site Description

**Figure 1:**
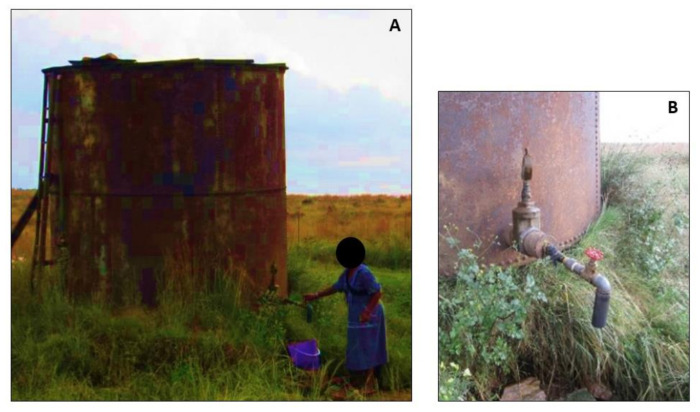
Central, large water storage tank (**A**), with close-up photo of tap (**B**).

**Figure 2:**
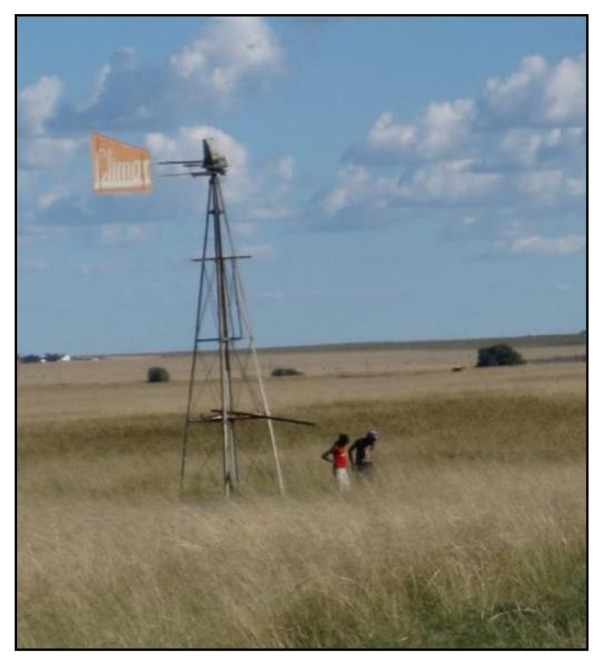
A tap connected to a windmill.

**Figure 3:**
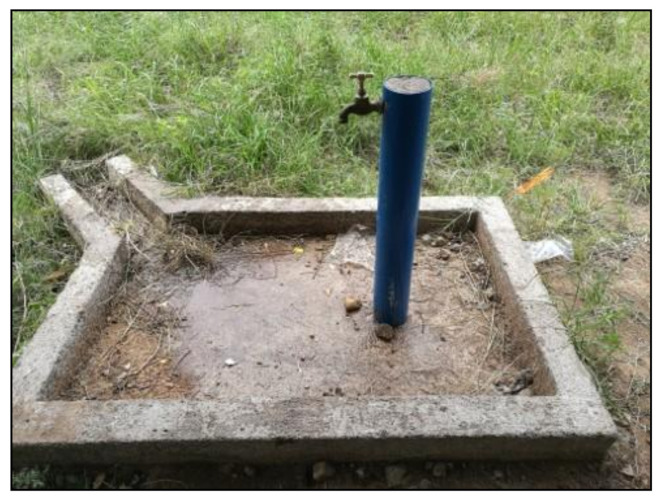
A communal tap outside the households.

Seven informal communities on the border between the Gauteng and Mpumalanga provinces in South Africa were selected as the study site. These communities all used borehole water as their primary drinking water source. The borehole water was collected by the households either from a large water storage tank (Figure 1), a tap connected to a windmill (Figure 2), or community taps outside the households in the street where one tap served about 5 households (Figure 3). In total, 82 households from these communities were included using a random selection method.

### 2.3. Point-of-Use Intervention

**Figure 4:**
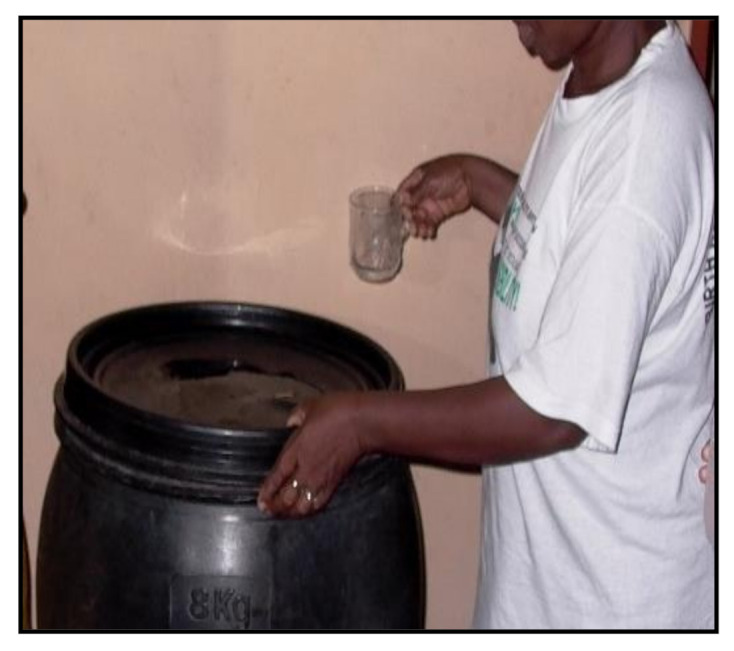
Jug used for water collection.

**Figure 5:**
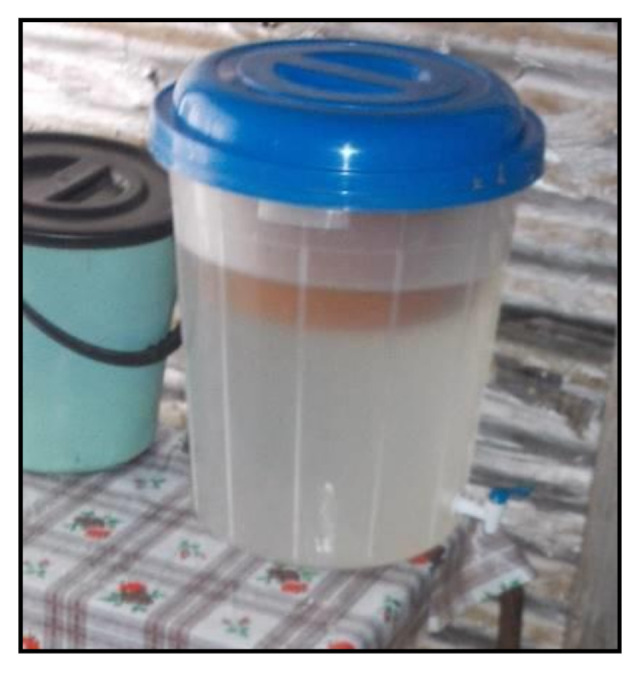
Ceramic pot device.

The intervention study was carried out over a period of five months (March to July) during 2016 and comprised of a ceramic pot filter device (CT Filtron) from Ceramica Tamakloe in Ghana. During the baseline (March and April), water samples were collected once a month from the 20-litre water storage container in all the households, which was used on the day of visitation as the container from which the household was using as drinking water and for cooking purposes to determine the level of microbial contamination prior to providing ceramic filtration units. After the baseline study, Potters for Peace (PFP) ceramic filter units were installed in all the households by members of the research team who explained to the female head of each household how to use, clean, and maintain the ceramic units. From May to July, water samples were collected once a month from the storage container in all households and from the ceramic unit’s filtered water. One litre of water (source and/or filter) was collected from each household in sterile plastic bottles, which was marked clearly with the name of the household and the sample type. The cup/jug (Figure 4) that the household used to decant water was used to collect water from the storage containers. When the research team collected filtered water from the ceramic unit, it was poured into the sterile collection bottle directly from the filter units’ spigot (Figure 5). The samples were kept on ice overnight and transported to the laboratory early the next morning for further assessment.

### 2.4. Water Quality Assessment Using E coli Bacteria Counts and Physical Parameters

Physical parameters included pH, electrical conductivity (EC), and turbidity (TDS), and readings of all water samples were measured onsite using a Crison Multimeter MM40 multi-meter (Crison, Allela, Spain). The probe was rinsed with distilled water before and after each measurement. All instruments were calibrated before use according to the manufacturer’s instructions. Microbiological quality of the water samples (1 L) was assessed using the Colilert^®^ Quanti-Tray/2000 system according to the manufacturer’s instructions and described by Omar et al. [53]. Briefly, a total of 100 mL of each water sample (undiluted) was tested, and the Quanti-Trays were incubated for 18 h at 35 °C. After incubation, the Quanti-Trays/2000 were examined under long wave (360 nm) ultraviolet light, and wells that turned yellow and fluorescent were counted as *E. coli* positive (IDEXX) and reported as per IDEXX tables as most probable number (MPN) count/100 mL.

### 2.5. Amoebal Enrichment of Water Samples

A total of 500 mL of each sample was filtered through a 0.45 µm nitrocellulose filter (Millipore, Burlington, MA, United States) with a filter manifold (Sartorius, Goettingen, Germany). The filter was aseptically placed face-side down onto a non-nutrient agar (NNA) plate and covered with a layer of heat-killed *Escherichia coli* (type strain, ATCC 25922), which served as a food source for the amoeba; then, a few drops of Paige’s amoebal saline (PAS) was added to aid in mobility, and the plates were incubated aerobically at 33 °C [6]. NNA plates were checked daily for the appearance of amoebal cysts using a 10× light microscope. A disposable Pasteur pipette was used to cut small plugs from the plates; these plugs were placed onto new NNA plates (covered with heat-killed *E. coli*), and one drop of PAS was added to each plug—this process purified the amoebae, as other organisms remained on the original plate. These were sub-cultured onto new NNA plates with *E. coli* and PAS until the culture was axenic. The sub-cultured plates were flooded with 2 mL of PAS, and a sterile plastic loop was used to gently scrape the axenic amoebae from the plate surface. This suspension was transferred to clearly marked, sterile 2 mL Eppendorf^®^ tubes. Before freezing the 2 mL tubes for later analysis, 1 mL of the amoebal suspension was lysed by passaging the suspension through a 27-gauge syringe and vortexed at 2500 rpm (Vortex Genie^®^ 2-Mixer 240 V, 50 Hz) to release the ARB. The lysed amoebae were stored in a different, sterile, marked tube and stored at −70 °C.

### 2.6. Isolation and Identification of FLA

Samples stored after initial amoebal enrichment were freshly inoculated onto NNA plates and allowed to grow. The plates were sealed individually with Parafilm M^®^ before it was packaged and sent to our collaborators at the Department of Hygiene, Social and Environmental Medicine (Ruhr-University Bochum, Bochum, Germany) for PCR and sequencing. Amoebal DNA was extracted from 200 μL of the prepared amoebae suspension in the amoebae-positive plates using the QIAamp DNA Blood Mini Kit (Qiagen, Hilden, Germany), according to the manufacturer’s protocol. The nucleic acid was eluted in 100 μL elution buffer into a sterile, clearly marked 1.5 mL microcentrifuge tube and stored at −20°C. Universal FLA genotyping was done using the forward primers Ami6F1 (5′-CCAGCTCCAATAGCGTATATT-3′), Ami6F2 (5′-CCAGCTCCAAGAGTGTATATT-3′), and reverse primer Ami9R (5′-GTTGAGTCGAATTAAGCCGC-3′) to amplify the 18S rRNA gene [6]. For *Acanthamoeba* genotype identification, amplification was done with the primer set JDP1 (5′-GGCCCAGATCGTTTACCGTGAA-3′) and JDP2 (5′-TCTCACAAGCTGCTAGGGGAGTCA-3′) [54]. Obtained sequences were aligned with sequences of *Acanthamoeba* genotypes T1–T20 [55] for identification.

### 2.7. Isolation and Identification of ARB

The amoebal enrichment suspensions were inoculated onto several different types of selective media for the isolation of specific bacteria. The study only focused on the following ARB species based on a seminal review article by Greub and Raoult [29]: *Salmonella*, *Shigella*, *Legionella*, *Mycobacterium*, *E. coli*, *Vibrio*, and Coliform bacteria. The selective media types used to isolate these bacteria included Brilliance^TM^ *E. coli*/coliform selective medium for *Escherichia coli* and other coliform bacteria (Oxoid—CM1046B); Xylose lysine deoxycholate (XLD) agar for *Salmonella* and *Shigella* spp. (Oxoid—CM0469B); *Thiosulphate Citrate Bile Salts Sucrose* (TCBS) agar for pathogenic *Vibrio* spp. (Oxoid—CM0333B); Charcoal yeast extract (CYE) agar (Oxoid—CM0655B) with *Legionella* buffered charcoal yeast extract (BCYE) growth supplement for *Legionellae* spp. (Oxoid—SR0110A); and Middlebrook 7H9 media with Oleic Albumin Dextrose Catalase (OADC) growth supplement for *Mycobacterium* spp. For each type of selective media, positive controls were used to ensure that the media would serve as an accurate source of information, regarding the unknown organisms cultured on it and included *Vibrio cholerae* O139 (NSCC), *Salmonella enteritidis*, (NSCC), *Shigella boydii* (NCTC 9329), *Escherichia coli* (ATCC 43888), *Legionella pneumophila* (ATCC 33152) and *Mycobacterium avium* (NSCC). All incubations were done according to specifications of the manufacturers. Further confirmation tests included the *Legionella* Latex Test (OXOID DR0800M, Hampshire, United Kingdom) to confirm *Legionella* species, and the Ziehl-Neelsen stain [56] to confirm *Mycobacterium* spp. Non-specific colonies on the selective media were picked and re-plated onto nutrient agar (Sigma-Aldrich—70148) to isolate single colonies for further identification. Likewise, single colonies identified by use of selective media as presumptive *Mycobacterium*, *Shigella*, *Legionella*, *Salmonella*, *Shigella*, *E. coli* or coliform species were also picked and streaked on nutrient agar for purification before molecular identification. All molecular identification was done by Inqaba Biotechnical Industries (Pty) Ltd. (Pretoria, South Africa). Only the 16S rRNA gene was investigated using primers sourced from the literature. These primers amplify almost the entire length of the gene [57], and the target region is conserved in many bacterial species [58]. For *Mycobacterium* spp. identification, the forward primer pA (5′-AGAGTTTGATCCTGGCTCAG-3′) and the reverse primer MSHE (5′-GCGACAAACCACCTACGAG-3′) were used [59]; for *Legionella* spp., the forward primer LEG 225 (5′-AAGATTAGCCTGCGTCCGAT-3′) and the reverse primer LEG 858 (5′-GTCAACTTATCGCGTTTGCT-3′) were used [60], and for the identification of the colonies from nutrient agar, XLD, and Brilliance *E. coli*/coliform media, the universal 16S rRNA primers pairs which included the forward primer 27 (5′-AGAGTTTGATCMTGGCTCAG-3′) and the reverse primer 1492 (5′-CGGTTACCTTGTTACGACTT-3′) were used [61].

## 3. Results

### 3.1. Collection of Water Samples

A total of 529 water samples consisting of 398 stored household water samples which were collected over the 5-month period and a total of 131 filtered water samples from the PFP ceramic pot filter which were collected over the 3-month intervention period were included in the study for assessment.

### 3.2. Water Quality Assessment Using Indicators

Table 1 provides the mean count (minimum to maximum counts) for the physical and the microbial indicator data for the stored water and filtered water samples. The pH of all water samples was within the South African Water Quality guideline standards for domestic use [62]. The average turbidity measurements were within the guideline value of 1 nephelometric turbidity units (NTU). However, some of the storage water samples had high NTU measurements, which indicated poor quality water. Turbidity can be caused by poor source water quality, and biofilms and high turbidity levels can protect microorganisms from the effects of disinfection and stimulate the growth of bacteria in stored water [63]. Electrical conductivity (EC) is a measure of the ability of water to conduct electricity, and this is directly dependent on the concentration of dissolved ions, which establishes a direct relationship between EC and total dissolved salts (TDS) [63]. In this study, the average EC measurements of filtered water samples were well within South African water quality guideline standard, but some of the water samples (stored and filtered) were above the South African water quality guideline limit of 170 millisiemens per centimeter (mS/cm) [62]. The presence of *E. coli* provides an indication of recent faecal contamination and should not be present in any water sample. The mean *E. coli* counts for household stored water were 173 MPN/100 mL, while the mean *E.* coli counts for filtered water were 23 MPN/100 mL (Table 1) which indicated faecal contamination [63].

### 3.3. Isolation and Identification of FLA

**Figure 6:**
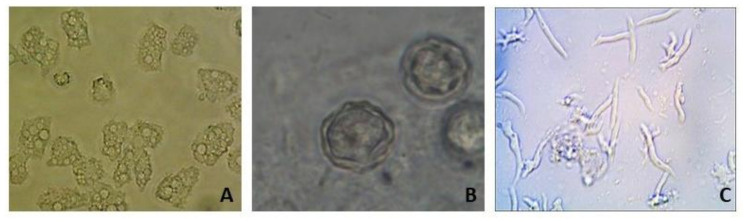
A 1000× magnification of (**A**) free-living amoebae (FLA) trophozoites; (**B)** double-walled *Acanthamoeba* spp. cysts, and (**C**) *Vermamoeba* spp. trophozoites.

Based on the morphological characteristics of trophozoites and cysts, several FLA were presumptively identified by light microscopy (Figure 6). Based on cyst morphology, 68 samples containing presumptive *Acanthamoeba* spp. from the household stored and ceramic pot filtered water samples were randomly selected and sent to our collaborators in Germany for 18S rRNA gene identification and sequencing. Results indicated that 20.6% of these samples were FLA negative; a total of 20.6% of the samples contained slime molds (*Mycetozoa* group); while *Vermamoeba vermiformis* was the most abundant amoebae identified, followed by *Acanthamoeba* spp. Other FLA identified included *Entamoeba nuttalli*, *Amoebozoa* ssp., *Stenamoeba* spp., *Flamella fluviatilis*, *Lobosea* spp., *Vexillifera westveldii*, and *Copromyxa protea* (Table 2). Co-occurrence of FLA species was identified in 45.6% of the samples.

### 3.4. Isolation and Identification of ARB

Contaminants such as hookworms and rotifers caused the exclusion of 76 samples from further testing, as an axenic culture could not be established, and only 453 water samples were assessed for the presence of ARB. The selective media for *Vibrio* spp., *Salmonella* spp., or for *E. coli* showed no growth, and growth on BCYE media could not be confirmed by the *Legionella* Latex test as *Legionella* spp. Table 3 provides a list of ARB isolates isolated and identified from water collected from the stored household container and the point-of-use ceramic pot filters. Species included several different *Achromobacter* spp., *Arthrobacter* spp., *Caulobacter* spp., *Enterobacter* spp., *Klebsiella* spp., *Microbacterium* spp., *Paenibacillus* spp., *Pragia* spp., *Pseudomonas* spp., *Rhodococcus* spp., *Serratia* spp., *Stenotrophomonas* spp., *Alcaligenes* spp., *Pseudomonas* spp., and non-tuberculous *Mycobacterium* spp. Some of these species have been identified as human pathogens while some are mainly found in the natural environment (water, soil, and plant samples).

## 4. Discussion

The objective of this study was to isolate and identify FLA and ARB in borehole water before and after a PFP ceramic pot filter intervention in rural communities. Genotyping identified many different amoebal species (e.g., *Flamella* spp., *Echinamoeba* spp., *Acanthamoeba* spp., *Vexillifera* spp., *Vermamoeba* spp., *Amoebazoa* spp., *Stenamoeba* spp., and *Vexillifera* spp.) present in storage and filtered water samples; consistent with previously isolated and reported FLA from different water sources (Table 2). Results have further shown that *V. vermiformis* was the most abundant FLA isolated from the water samples. Several studies have shown that *V. vermiformis* is not just a pathogen on its own, it has also been associated with numerous ARB species, including *Mycobacteriae* [157,158,159,160], *Pseudomonas aeruginosa* [161], and *S. maltophilia* [77,161], amongst others. A study by Scheid [31] detected *V. vermiformis* when examining a patient with *Acanthamoeba keratitis*, which in one case caused an ulcer on a human eyelid.

The *Acanthamoeba castellanii* genotype T4 is reported in the literature to be the most frequently isolated genotype in human infections [28,162]; it accounts for more than 90% of cutaneous GAE and *A. keratitis* infections [28]. According to Siddiqui and Khan [163], this genotype is more virulent and more likely to be transmitted than other genotypes, and they are also less susceptible to disinfection processes. Cases of AK caused by genotypes T3 and T4 have been reported in Mexico [164], T4 and T15 have been reported in Italy [165], and genotype T16 has been recovered from bromeliads in Brazil [166]. The only *Entamoeba* spp. isolated and identified in this study was *Entamoeba nuttalli*, which is a known monkey pathogen [167] and has been isolated from the faecal samples of rhesus macaques and marmoset monkeys [71,168]. Several vervet monkeys (*Chlorocebus pygerythrus*) were often sighted in the study area and could be the reason for the strain’s presence in the borehole water (Table 2). There were also many slime molds recovered from the samples (Mycetozoa group). The probable reason why organisms from the group Mycetozoa were isolated by the same means as the FLA could be because Mycetozoa forms part of the super-group Amoebozoa, which consists of numerous amoebae [169]. A study by Denoncourt et al. [170] also showed that slime molds could harbour bacteria. Future studies should investigate these organisms when looking for intra-amoebal bacteria.

Well-known ARB species such as *Vibrio* spp., *Salmonella* spp., *Legionella* spp., or *E. coli* were not isolated and identified via amoebal enrichment, although indicator tests on the samples were positive for *E. coli*. (Table 3). However, during FLA identification in Germany, six samples of *Legionella* spp. were identified by PCR and sequencing in the samples send (data not shown). In one of the samples, *L. pneumophila* co-occurred with *V. vermiformis*, while in the other samples, *L. pneumophila* co-occurred with other FLA species. According to Buse et al. [171], *L. pneumophilia* survival is impacted by FLA predation together with secreted molecule(s) from the amoeba, which inhibits the bacteria’s growth and stimulates the survival of the *Legionella* bacteria by inducing the viable but not culturable (VBNC) state and inhibits the infectivity of the *Legionella* bacteria and their interactions with other FLA hosts.

Nevertheless, other ARB species were isolated and identified from the water and included *Mycobacterium* spp., *Pseudomonas* spp., *Stenotrophomonas* spp., *Serratia* spp., *Pragia* spp., *Rhodococcus* spp., *Paenibacillus* spp., *Microbacterium* spp., *Caulobacter* spp., *Klebsiella* spp., *Enterobacter* spp., *Arthrobacter* spp., and *Achromobacter* spp. (Table 3). Many of these ARB species have previously been associated with clinical infections ranging from bacteraemia, pneumonia, eye infections, and meningitis, to infections caused by bacterially colonised implanted medical devices; thus, they pose a serious risk (potentially fatal at times) to human health with the regular use and consumption of such water sources (Table 3).

The indicator *E. coli* counts were used as the norm to indicate the effectivity of the point-of-use PFP filter, and Table 1 showed that the filters removed the indicator bacteria from the borehole water. However, the average filter performance of the Ghana ceramic pot filters did not meet the recommended 2 log10 reduction for home treatment [172]. The PFP ceramic filters are manufactured in different low-income countries, and although the design is the same, the manufacturing processes and the materials used have an impact on the efficacy of the filters [172,173,174]. Van Halem [175] tested the Ghanaian filters and found that they were rough (due to a protective layer in the press mould), with shapes that were not identical, and the rims of the filters were not always straight. The Ghanaian filters also had fluxes greater than the 2 litres per hour boundary, which indicates a flow rate not compatible with the removal of microorganisms [176]. According to Lantagne [52], the pore size of the ceramic filters should be approximately 0.6 to 3 µm. The average size of amoebae ranges from 7 µm (*N. fowleri* cyst) to 80 µm (*S. pedata* trophozoite) [177]. However, cracks in the ceramic could create spaces with lengths of between 150 and 500 µm [177]. Some of the ARB could have been protected by the FLA and went through the cracks into the filtered water where they were detected. This may explain why some bacteria were found in the filtered water. It is also possible that the filters were contaminated by poor hygiene conditions or some of the filters had allowed the bacteria through because of the cracks from during the manufacturing of the pots [172]. The filter receptacle could also have been contaminated by incorrect use by the household members; it was observed during this study that even though users of the filters were trained on how to properly care for their filter, a few receptacles were not cleaned as instructed and washed with unclean water, and uncleaned cloths were used. In addition, lysing of FLA to release the ARB can happen at any given time, usually when conditions become more favourable [7,29]. A study by Yu et al. [178] proved that *Mycobacterium* spp. can be propagated in protozoa for more than six years, and neither organism will show ill effects.

Studies have shown that FLA and ARB are resistant to conventional disinfectants used in drinking water treatment [179]. No study could be found investigating the effect of colloidal silver (which is painted on the inside of ceramic filters) on FLA and ARB species, although several studies did report on the effect of heat, heat shock treatment, disinfections such as chlorine, bromine, isothiazolinone, peracetic acid (PAA) mixed with hydrogen peroxide, and innovative enzymatic treatment using proteins such as subtilisin on FLA species [180,181,182]. Newer technologies such as silver nanoparticles conjugated with oleic acid have shown anti-amoebal effects against both *A. castellanii* and *N. fowleri* during in vitro studies and may be a future solution in lowering the number of FLA in point-of-use technologies for safe drinking water [183,184]. Therefore, more studies are needed to assess the effect of disinfection solutions on FLA and ARB species.

Limitations of the study included the following: (a) no clinical samples were collected to confirm whether the organisms isolated have been associated with infections caused in humans; (b) not all the samples could be sequenced due to a very limited budget for molecular studies and sequencing; and (c) many of the samples also contained hookworms and rotifers and could not be further assessed. Several studies have shown that hookworms are present in the faeces of livestock [185] and domestic animals [186], and therefore, the eggs could have entered underground aquifers, while several reports on the prevalence of rotifers in groundwater have been published [187,188]. Hence, more studies are needed to determine the health risk to vulnerable individuals drinking water containing these organisms and the effectiveness of point-of-use devices in eliminating them. Nevertheless, the study has shown that potentially harmful FLA and ARB species are present in borehole water, and if point-of-use devices do not remove these organisms efficiently, these organisms can be a health risk for vulnerable individuals in the community.

## 5. Conclusions

This is the first study in a South African rural setting that reports on the isolation and prevalence of FLA and ARB in borehole water, both before and after a point-of-use intervention. It can be presumed, based on other reported studies, that the FLA and ARB species identified in this study might have the potential to cause diseases in both immunocompromised and immunocompetent individuals. In some cases, the amoebae itself may be quite harmless, but they could shelter potential pathogenic bacteria. Therefore, it is important not to only use *E. coli* as a reference on the efficiency of ceramic pot filters for household drinking water treatment. Further in-depth studies will be needed to (1) monitor the prevalence and co-occurrence of FLA and ARB in water for human consumption and (2) test the effectiveness of different ceramic pot filtration systems in removing FLA and ARB from drinking water.

## Figures and Tables

**Table 1:** Average counts for indicators of water samples.

Indicator	Household Storage Water	Household Filtered Water	South African Water Quality Guideline [62]
*E. coli* count (MPN/100 mL)	173 (<1–>2419.6)	23 (<1–>2419.6)	0
pH	8.25 (5.65–9.29)	8.11 (6.87–9.18)	≥5.0–≤9.7
Turbidity (NTU)	0.66 (0.11–18.4)	0.46 (0.17–2.99)	1
Conductivity (mS/cm)	91.73 (18.5–185.3)	74.11 (17.2–221.9)	170

MPN: most probable number; NTU: nephelometric turbidity units; mS: millisiemens.

**Table 2:** FLA isolated and identified from household storage container water and the ceramic pot water sources, and information where the strain has been isolated in other reported studies (*n* = total number of samples identified with this spp).

FLA Species	*n*	Stored Water	Filtered Water	Published Studies Indicating Where the Organism Was Previously Isolated with References
***Amoebozoa* spp.**	**5**	3	2	Marine-/fresh water and soil [64]; Groundwater [65]
***Acanthamoeba* spp.**	**2**	2	0	Soil [66]
Genotype T3	**1**	1	0	Corneal biopsy of contact lens wearer [67]
Genotype T4	**2**	2	0	Corneal scrape of contact lens wearer [67]
Genotype T15	**1**	1	0	Corresponding to only *A. jacobsi* [25,68]
Genotype T16	**2**	2	0	Freshwater pond, Italy [26]
*A. castellanii* str. Neff	**3**	2	1	Soil [66]
***Echinamoeba* spp.**	**1**	0	1	Hot water springs [69]
*E. exudans*	**2**	0	2	Leaf litter [70]
***Entamoeba* spp.**				
*E. nuttalli*	**3**	2	1	Rhesus macaque [71]
***Flamella* spp.**	**2**	2	0	Depends on species [72]
*F. beringiania*	**1**	1	1	Siberian permafrost [73]
*F. fluviatalis*	**1**	1	0	Fresh water [74]
* Hartmanella spp.	**2**	1	1	Potable water supplies [75]
***Stenamoeba* spp.**	**2**	2	0	Freshwater lake sediment [76]
***Vermamoeba* spp.**				
*V. vermiformis*	**34**	17	17	Variety of water sources [77,78]; bat guano [79]
***Vexillifera* spp.**				
*V. westveldii*	**2**	1	1	Pond water [80]

* *Hartmanella* spp. are now called *Vermamoeba* spp. [31].

**Table 3:** Amoeba-resistant bacteria (ARB) isolated and identified from household storage container water and the ceramic pot water sources, and information where the organism has been isolated in other reported studies (*n* = total number of samples identified with this species).

ARB Species	*n*	Stored Water	Filtered Water	Published Studies Indicating Where the Organism Was Previously Isolated with References
***Achromobacter* spp.**	**4**	3	1	Freshwater; sea water, soil [81]
*A. insolitus*	**1**	1	0	Laboratory sink, urine, wounds [82]
*A. marplatensis*	**1**	1	0	Pentachlorophenol-contaminated soil [83]
*A. spanius*	**2**	1	1	Human clinical samples [82]
*A. xylosoxidans*	**1**	0	1	Oligotrophic aquatic niches; opportunistic pathogen; human bacteremia; pneumonia [84,85]
***Arthrobacter* spp.**				
*A. nicotinovorans*	**1**	1	0	Soil [86]
***Caulobacter* spp.**				
*C. segnis*	**1**	1	0	Soil [87]
***Enterobacter* spp.**				
*E. amnigenus*	**1**	1	0	Mallard duck intestines; parasitic disease caused by Kenyan sand fly [88,89]
*E. asburiae*	**6**	6	0	Human clinical samples [90]
*E. cancerogenus*	**2**	2	0	Human clinical samples [91]
*E. kobei*	**2**	2	0	Implanted medical devices; nosocomial bacteria in ICU patients [92,93]
*E. ludwigii*	**8**	6	2	Human clinical samples [92]
***Klebsiella* spp.**				
*K. oxytoca*	**1**	1	0	Mammal mucosal surfaces; hospital-acquired pathogen; various human infections [94,95,96]
*K. variicola*	**1**	1	0	Environmental samples [97,98,99,100]
***Microbacterium* spp.**	**1**	1	0	Human clinical samples [101]
*M. oxydans*	**4**	3	1	Human clinical samples [101]
*M. paraoxydans*	**12**	6	6	Human clinical samples [101]
***Paenibacillus* spp.**				
*P. validus*	**1**	1	0	Soil, water, rhizosphere, vegetable matters, fargae, insect larvae and human clinical samples [102,103]
***Pragia* spp.**				
*P. fontium*	**1**	1	0	Water wells; pipes [104]
***Pseudomonas* spp.**	**4**	1	3	Saprophytes; contaminated human clinical samples [105]
*P. fluorescens*	**7**	3	4	Plants, soil, rhizosphere, human clinical samples [106,107,108,109]
*P. geniculata*	**1**	1	0	Refrigerated meat; dairy products; maple tree sap [110,111,112]
*P. kilonensis*	**1**	1	0	Agricultural soil [113]
*P. koreensis*	**2**	1	1	Agricultural soil [114]
*P. monteilii*	**1**	0	1	Human clinical samples [115]
*P. moraviensis*	**1**	0	1	Soil [116]
*P. otitidis*	**1**	0	1	Clinical specimen—human ears [117]
*P. poae*	**1**	1	0	Phyllosphere of grasses [118]
*P. rhodesiae*	**1**	0	1	Natural mineral water [119]
*P. tremae*	**1**	1	0	Plants [120]
*P. vancouverensis*	**1**	1	0	Soil [121]
*P. putida*	**3**	1	2	Plants, soil, rhizosphere, human clinical samples [106,107,108,109]
***Rhodococcus* spp.**				
*R. erythropolis*	**1**	1	0	Soil, rocks, groundwater, seawater, plants, animals, gut of insects [122]
***Serratia* spp.**				
*S. ureilytica*	**1**	1	0	River water [123]
***Stenotrophomonas* spp.**				
*S. maltophilia*	**8**	4	4	Human pathogen; humid surfaces; medical devices [124,125,126]
*S. rhizophila*	**1**	0	1	Plant-associated [127,128]
*Alcaligenes faecalis and Achromobacter marplatensis*	**1**	1	0	*A. faecalis*—faeces, soil, water, other environments [129,130,131];
				*A. marplatensis*—Pentachlorophenol-contaminated soil [83]
***Pseudomonas* spp.**				
*P. poae/P. tolaasii*	**1**	1	0	Phyllosphere of grasses [118]; compost; casting soil in mushroom production [132]
*P. fluorescens/P. rhodesiae*	**1**	0	1	Plants, soil, rhizosphere; human clinical samples [106,107,108,109]; Natural mineral water [120]
*Glutamicibacter uratoxydans* and *Stenotrophomonas maltophilia*	**2**	0	2	*G. uratoxydans* (previously known as *Arthrobacter uratoxydans*) in humus soil [133]; *S. maltophilia*—Human pathogen; humid surfaces; medical devices [124,125,126]
***Mycobacterium* spp.**				
*M. chlorophenolicum*	**1**	1	0	Soil; sludge; Pneumonia [134,135]
*M. chubuense*	**4**	3	1	Water; soil [136,137]
*M. cosmeticum*	**1**	0	1	Nail salons [138]
*M. elephantis*	**1**	1	0	Elephant carcass [139]
*M. fallax*	**1**	1	0	Water [140]
*M. farcinogenes*	**1**	1	0	Bovine farcy [141]
*M. florentinum*	**10**	8	2	Sputum; lymph node [142]
*M. gilvum*	**1**	1	0	Water; soil [143]
*M. intermedium*	**5**	4	1	Sputum [144]
*M. llatzerense*	**7**	4	3	Hemodialysis water [145]
*M. noviomagense*	**1**	1	0	Respiratory samples [146]
*M. pallens*	**3**	3	0	Soil [147]
*M. poriferae*	**7**	6	1	Treatment plants [148,149]
*M. psychrotolerans*	**1**	1	0	Water near uranium mine [150]
*M. rhodesiae*	**1**	1	0	Human clinical samples; soil [151]
*M. salmoniphilum*	**15**	15	0	Salmonid fishes [152]
*M. smegmatis*	**1**	1	0	Environmental samples; rare human pathogen [151,153,154]
*M. tokaiense*	**1**	1	0	Soil [155]
*M. triplex*	**3**	2	1	Human pulmonary mycobacteriosis [156]

## Data Availability

Raw data is available by reasonable request from corresponding author.
